# Amino acids as regulators of gene expression

**DOI:** 10.1186/1743-7075-1-3

**Published:** 2004-08-17

**Authors:** Scot R Kimball, Leonard S Jefferson

**Affiliations:** 1 Department of Cellular and Molecular Physiology, The Pennsylvania State University College of Medicine, Hershey, PA 17033, USA

## Abstract

The role of amino acids as substrates for protein synthesis is well documented. However, a function for amino acids in modulating the signal transduction pathways that regulate mRNA translation has only recently been described. Interesting, some of the signaling pathways regulated by amino acids overlap with those classically associated with the cellular response to hormones such as insulin and insulin-like growth factors. The focus of this review is on the signaling pathways regulated by amino acids, with a particular emphasis on the branched-chain amino acid leucine, and the steps in mRNA translation controlled by the signaling pathways.

## Introduction

Recent advances in biomedical research reveal a key role for amino acids as nutritional signals in the regulation of a number of cellular processes. Studies employing a variety of cell types and different tissues demonstrate that one such process affected is the regulation of gene expression through modulation of the translation of messenger RNA (mRNA). The studies show that cells recognize changes in amino acid availability and generate alterations in signal transduction pathways that are also regulated by hormones and growth factors. The cells then respond to the integrated signaling input by either upregulating or downregulating translation initiation, i.e., the process during which initiator methionyl-tRNA (met-tRNA_i_) and mRNA bind to a 40S ribosomal subunit followed by the joining of a 60S ribosomal subunit to form a translationally competent 80S ribosome. The response of translation initiation to a change in amino acid and/or hormone availability can be general, i.e., affecting the translation of most if not all mRNAs, and/or specific, i.e., affecting the translation of a single class or subset of mRNAs. Both the general and specific responses can be mediated through regulation of either the met-tRNA_i _and/or mRNA binding steps. The specific response may also involve an additional regulatory site, i.e., the phosphorylation status of ribosomal protein rpS6, one of the proteins composing the 40S ribosomal subunit. Learning how the cell recognizes a sufficiency of amino acids is presently the objective of intense research. Present evidence, however, suggests multiple recognition sites and multiple signaling pathways. Below, we summarize our current knowledge of the signaling pathways known to respond to changes in amino acid availability. In addition, the translation initiation factors and mRNA structural elements that are involved in changes in both global and specific modulation of mRNA translation are discussed.

## mRNA translation initiation

The first step in translation initiation involves the binding of met-tRNA_i _to the 40S ribosomal subunit, a reaction mediated by the eIF2•GTP complex [reviewed in [1]]. In a subsequent step, the GTP bound to eIF2 is hydrolyzed to GDP and eIF2 is released from the 40S subunit complexed with GDP, leaving met-tRNA_i _behind. Exchange of GDP bound to eIF2 for GTP is mediated by the guanine nucleotide exchange factor eIF2B, and as described below, there are at least three known mechanisms for modulating eIF2B activity in vivo.

The second step in translation initiation involves the binding of mRNA to the 40S ribosomal subunit containing the eIF2•GTP•met-tRNA_i _complex and eIF3 [1]. The protein that mediates this step is a heterotrimeric complex referred to as eIF4F which consists of the initiation factors eIF4A, eIF4E, and eIF4G. eIF4A is an RNA helicase that serves to unwind secondary structure in the 5'-untranslated region (5'-UTR) of the mRNA, allowing the 40S ribosomal subunit to migrate from the 5'-m^7^GTP cap to the AUG start codon. The helicase activity of eIF4A is stimulated by eIF4B and eIF4H. eIF4E binds to the m^7^GTP cap at the 5'-end of the mRNA and thus plays a crucial role in the binding of the mRNA to the ribosome. eIF4G is a scaffolding protein that binds to eIF4A, eIF4E, and eIF3. Thus, eIF4G is a molecular bridge that links the mRNA, which is bound by eIF4E, to the 40S ribosomal subunit, which is bound by eIF3. Assembly of the eIF4F complex is regulated in part through the reversible association of eIF4E with the translational repressors, eIF4E-binding proteins 4E-BP1, 4E-BP2, and 4E-BP3. The domain on eIF4E to which eIF4G binds overlaps with the binding domain for the 4E-BPs, such that either eIF4G or 4E-BP can bind to eIF4E, but both cannot bind at the same time. Thus, association of eIF4E with a 4E-BP precludes the binding of mRNA to the 40S ribosomal subunit by preventing the binding of the eIF4E•mRNA complex with eIF4G. Association of eIF4E with the 4E-BPs is regulated by phosphorylation of 4E-BP, whereby hypophosphorylated 4E-BPs bind to eIF4E but the hyperphosphorylated proteins do not.

## Regulation of mRNA translation through Phosphorylation of eIF2 or eIF2B

**Figure 1:**
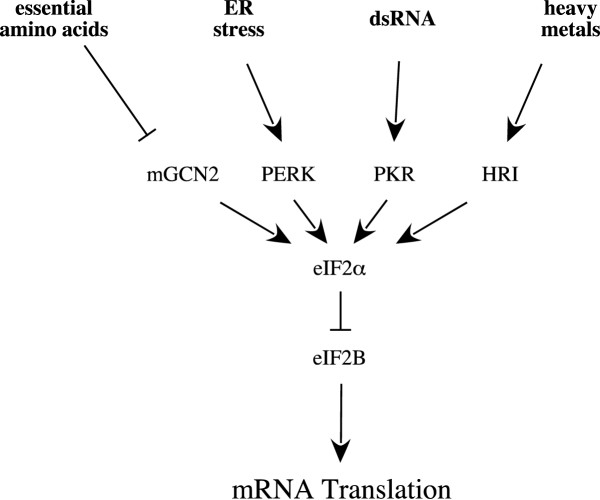
Regulation of eIF2α phosphorylation. Phosphorylation of eIF2α is mediated by four known protein kinases that are regulated by diverse cellular stresses. Phosphorylation of eIF2α inhibits eIF2B which can have both general and specific effects on mRNA translation as described in detail in the text.

Of the three known mechanisms for regulating eIF2B activity, the best characterized involves phosphorylation of eIF2 on Ser51 of its α-subunit. Phosphorylation of eIF2α converts eIF2 from a substrate into a competitive inhibitor of eIF2B and represses the translation of most mRNAs, but paradoxically enhances the translation of mRNAs containing multiple upstream open reading frames (uORF) and internal ribosome entry sites (IRESs). Phosphorylation of eIF2α is mediated by any of four known eIF2α kinases in mammalian cells: the mammalian ortholog of the yeast general control non-derepressing kinase-2 (mGCN2), the heme-regulated inhibitor (HRI), the protein kinase dsRNA-activated (PKR), and the PKR-like endoplasmic reticulum kinase [PERK, reviewed in [2]] (Fig. 1). In both cells in culture [e.g. [3]] and livers perfused in situ [4], deprivation of single essential amino acids promotes phosphorylation of eIF2α with a concomitant inhibition of eIF2B. The phosphorylation of eIF2α that occurs in vivo [5], in perfused rat liver [6], and in cells in culture [7] in response to altered amino acid availability is mediated by the eIF2α protein kinase referred to as mGCN2. In yeast deprived of amino acids, uncharged tRNA accumulates and binds to a domain on Gcn2p that exhibits sequence homology to histidyl-tRNA synthetase resulting in its activation [reviewed in [5]]. In fasted rats, feeding a meal containing a complete mixture of essential amino acids stimulates protein synthesis in the liver and skeletal muscle, but has no effect on eIF2α phosphorylation or eIF2B activity [8]. In contrast, feeding a diet lacking a single essential amino acid results in both an increase in eIF2α phosphorylation and a reduction in eIF2B activity in liver [9], suggesting that an imbalance in plasma concentrations of essential amino acids results in activation of signaling pathways within the liver that result in increased phosphorylation of eIF2α. The enhanced phosphorylation of eIF2α that occurs in response to an imbalanced amino acid mixture is mediated by the eIF2α kinase mGCN2, because in mice lacking the kinase, feeding a diet lacking leucine does not promote eIF2α phosphorylation or inhibition of eIF2B [5]. However, the mechanism through which severe amino acid deprivation activates mGCN2 in cells in culture, i.e. accumulation of uncharged tRNA, probably isn't relevant in vivo because plasma amino acids are typically maintained at concentrations well above the K_m _of the aminoacyl-tRNA synthetases, even during fasting, and therefore significant amounts of uncharged tRNA are unlikely to accumulate. Modulation of eIF2α phosphorylation also occurs in response to changes in the availability of nutrients other than amino acids. For example, either hypoglycemia or hyperglycemia promotes eIF2α phosphorylation. Hypoglycemia is thought to activate the endoplasmic reticulum-associated eIF2α kinase termed PERK through induction of the ER stress response [10]. However, the kinase that phosphorylates eIF2α in response to hyperglycemia is unknown. In vivo, the transient hypoglycemia that occurs shortly after birth results in altered translation of mRNAs encoding several transcription factors such as C/EBPβ that induce the transcription of a number of genes involved in gluconeogenesis and glucose storage such as PEPCK, glucose-6-phosphatase, pyruvate carboxylase, and glycogen synthetase [11]. A recent study using mice containing a homozygous mutation in the gene encoding eIF2α that replaces Ser51 with an unphosphorylatable Ala residue (eIF2S51A) demonstrated that phosphorylation of eIF2α is a critical component in the response of the newborn to hypoglycemia [12]. Thus, in neonatal eIF2S51A mice, the activity of PEPCK in the liver is significantly reduced compared to wildtype mice and its induction immediately after birth is severely attenuated. The mechanism through which eIF2α phosphorylation might promote induction of PEPCK gene transcription is as yet unexplored, but has been postulated to be a consequence of altered translation of mRNAs encoding specific transcription factors, e.g. C/EBPα and C/EBPβ.

HRI was first identified in rabbit reticulocytes and shown to be activated in response to hyperoxia and iron and heme deficiency [13]. Subsequent studies have shown that HRI is also expressed in multiple tissues and is activated by heavy metals and nitric oxide (NO). In fact, NO binds directly to HRI [14]. Because non-erythroid cells seldom experience large fluctuations in heme content, it has been suggested that NO may be a principle regulator of HRI in such cells [14].

PKR is a ubiquitously expressed serine-threonine protein kinase that is activated by double-stranded RNA and is induced by interferon [reviewed in [15]]. PKR is also activated by lipopolysaccharide and cytokines such as IL-1 and TNF-α, and is a key component of the proinflammatory response to bacterial infection. It is a potent inhibitor of cell growth when over-expressed in yeast, mammalian, or insect cells, an effect that is mediated by eIF2α phosphorylation because co-expression of a non-phosphorylatable eIF2α prevents the growth repressive effect [16]. Unlike the other three eIF2α kinases, eIF2α is not the only substrate for PKR; for example, PKR is reported to phosphorylate the regulatory subunit of protein phosphatase 2A [17]. PKR also binds to the IκB kinase complex and is involved in NF-κB signaling.

In addition to changes in eIF2α phosphorylation, eIF2B activity can be altered through changes in expression of the catalytic ε-subunit. Knockdown of the catalytic ε-subunit using RNA_i _essentially halts cell growth and triggers apoptosis [18]. In contrast, overexpression of eIF2Bε, as occurs in many transformed cells, results in increased growth [19]. Because the ε-subunit alone is not inhibited by phosphorylated eIF2, overexpression of eIF2Bε provides a means of enhancing mRNA translation under stress conditions that promote eIF2α phosphorylation. The mechanism(s) through which eIF2Bε expression is regulated are unknown, but our laboratory has found that a preferential increase in eIF2Bε expression occurs in response to acute resistance exercise and is blocked by pre-treatment with rapamycin, a specific inhibitor of the mammalian target of rapamycin (mTOR) (unpublished observation). Because both nutrients and growth-promoting hormones stimulate the mTOR signal transduction pathway (see the next section for further discussion of mTOR signaling), it is tempting to speculate that expression of eIF2Bε might be enhanced by such stimuli.

The guanine nucleotide exchange activity of eIF2B may also be subject to regulation through phosphorylation of its ε-subunit. In vitro, at least four kinases phosphorylate eIF2Bε including casein kinases (CK)-I and -II, glycogen synthase kinase (GSK)-3, and DYRK. Phosphorylation of eIF2Bε by either CK-I [20] or CK-II [20,21] reportedly stimulates the activity of eIF2B, although this conclusion has been questioned by another group [22]. Whether or not phosphorylation by GSK-3 alters the activity of eIF2B is likewise controversial. One study [20] reports that phosphorylation by GSK-3 has no direct effect on eIF2B activity, even though phosphorylation by GSK-3 prevents the subsequent phosphorylation, and thus activation, by CK-I. In contrast, other studies suggest that phosphorylation of Ser535 in rat eIF2Bε (Ser540 in the human sequence) by GSK-3 is required, but not sufficient, for inhibition of eIF2B activity by insulin [23].

## Regulation of mRNA translation through downstream targets of the mTOR signaling pathway

**Figure 2:**
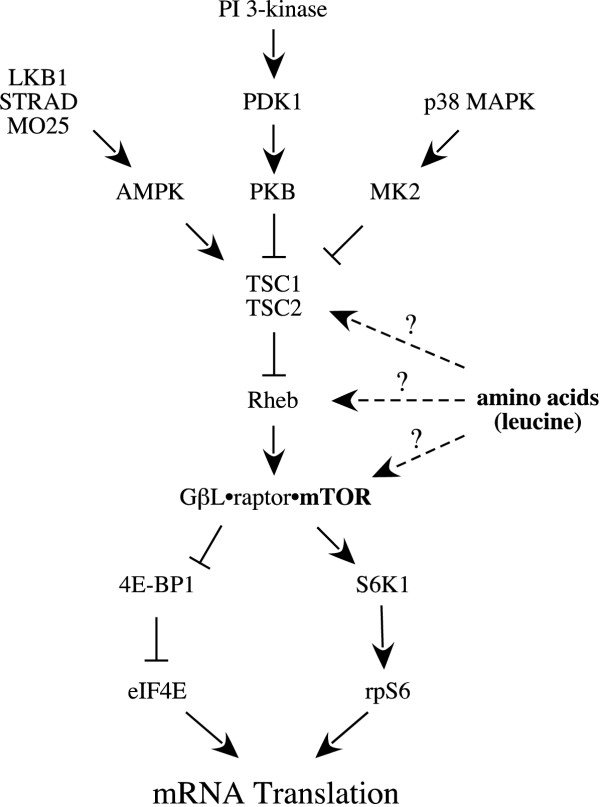
Regulation of the mTOR signaling pathway. The mTOR signaling pathway is controlled through various upstream kinases (e.g. AMPK, AKT, and MK2) that converge on the tuberous sclerosis complex, TSC1•TSC2. TSC2 is a GTPase-activator protein for Rheb which is a positive effector of signaling through mTOR. mTOR signals to downstream targets such as 4E-BP1 and S6K1 as a complex with the regulatory proteins raptor and GβL as described in detail in the text.

The protein kinase mTOR is a common intermediate in both nutrient and hormone signal transduction pathways (Fig. 2). Signaling through mTOR is enhanced by nutrients and anabolic hormones, such as insulin or IGF-I [24,25], and repressed by elevation of cAMP [25-27] or activation of AMPK [28-30], suggesting that one function of mTOR is to integrate the anabolic response to nutrients and insulin and the catabolic response to counter-regulatory hormones, such as glucagon. However, mTOR may not be a direct target of nutrient and hormone signaling. Instead, a number of recent studies have identified TSC1•TSC2 as a potential branch point in the nutrient, insulin, and AMPK signaling pathways to mTOR [31,32]. The results of these studies support a model wherein insulin and leucine would repress the inhibitory action of TSC1•TSC2 on mTOR signaling whereas glucagon would stimulate it. In this model, insulin stimulates signaling to mTOR through Akt-mediated phosphorylation of TSC2. Leucine would also modulate signaling through mTOR through the TSC1•TSC2 complex. However, the mechanism through which leucine signals to TSC1•TSC2 is unknown, but is distinct from Akt. Leucine may also modulate signaling through mTOR by altering the association of the kinase with one or more regulatory proteins, such as the regulatory associated protein of mTOR (raptor) and G protein β-subunit-like protein (GβL). In the paragraphs that follow, the evidence supporting these various mechanisms for regulating mTOR is discussed.

The activity of mTOR toward downstream targets such as 4E-BP1 and S6K1 is controlled in part through the interaction of mTOR with the regulatory proteins raptor and GβL. Evidence linking raptor with nutrient signaling through mTOR is provided by studies wherein raptor expression was downregulated using siRNA [33,34]. In such studies, leucine-induced phosphorylation of S6K1 is greatly repressed to an extent similar to that observed in cells in which mTOR expression is reduced. In part, leucine may modulate signaling through mTOR by altering the stability of the mTOR•raptor complex. In this regard, in one study the stability of the mTOR•raptor complex was found to be enhanced in cells subjected to amino acid deprivation [33]. However, a study by another group [35] failed to observe a change in binding of raptor to mTOR in cells starved for amino acids. In part, this discrepancy may be explained by the identification of GβL as a second mTOR-interacting protein [34]. Like raptor, GβL has been shown to co-immunoprecipitate with mTOR [34]. GβL is a positive regulator of mTOR because co-expression of GβL with mTOR results in greatly increased kinase activity of mTOR toward 4E-BP1 and S6K1 compared to expression of mTOR alone [34]. Moreover, reducing GβL expression using siRNA represses leucine- and serum-induced phosphorylation of S6K1 [34], suggesting that GβL is involved in hormone and amino acid signaling though mTOR. Importantly, GβL is necessary for leucine-mediated changes in mTOR•raptor association. In cells deprived of leucine, the binding of both raptor and GβL is high and readdition of leucine to leucine-deprived cells decreases the amount of raptor, but not GβL, associated with mTOR [34]. However, leucine-induced changes in mTOR•raptor association requires GβL, suggesting that the binding of GβL to mTOR renders the binding of raptor to mTOR sensitive to changes in amino acid availability.

The most proximal upstream protein that has been identified in the mTOR signaling pathway is the Ras homolog enriched in brain (Rheb). Rheb is a small G protein that enhances phosphorylation of S6K1, rpS6, and 4E-BP1 in an mTOR-dependent fashion when overexpressed [reviewed in [31,36]]. Moreover, in cells overexpressing Rheb, S6K1 phosphorylation is maintained during starvation for amino acids, suggesting that Rheb is involved in transducing signals from amino acids through mTOR [37,38]. Rheb activity is controlled in part by a GTPase activating protein (GAP) referred to as TSC2 or tuberin. TSC2, and its binding partner TSC1 (a.k.a. harmartin) were originally identified as the product of two genes that are causative in the autosomal dominant syndrome tuberous sclerosis [reviewed in [39-43]]. Mutations in either gene are associated with the widespread development of benign growths in multiple organs and tissues, suggesting that the normal role of these proteins is to restrict cell size and proliferation. This idea has been confirmed in studies in which the Drosophila orthologs of TSC1 and TSC2, dTsc1 and dTsc2, respectively, were shown to function in a complex that acts downstream of AKT but upstream of Drosophila TOR (dTOR) to restrict cell growth and proliferation [44-46]. Studies in both Drosophila [47] and mammalian cells [48] have implicated TSC1 and TSC2 in amino acid signaling through TOR. In Drosophila, downregulated expression of either protein causes cells to become resistant to amino acid deprivation [47]. Thus, S6K phosphorylation is largely maintained during amino acid starvation in cells with reduced expression of either dTsc1 or dTsc2 [47]. Similarly, in mammalian cells lacking either TSC1 or TSC2, S6K1 phosphorylation is resistant to amino acid deprivation [49]. Moreover, in mammalian cells in culture, co-overexpression of TSC1 and TSC2 prevents amino acid-dependent activation of S6K1 [48]. Together, these studies strongly suggest that TSC1 and TSC2 are required for amino acid induced signaling through mTOR.

The mechanism(s) involved in the regulation of TSC2 GAP activity are poorly understood, but likely involve phosphorylation of the protein by multiple upstream protein kinases. For example, TSC2 has been shown to be directly phosphorylated by AKT on multiple serine and threonine residues and phosphorylation by AKT represses the inhibitory action of the TSC1/TSC2 complex on signaling through mTOR to 4E-BP1 and S6K1 [50-53]. Likewise, phosphorylation of TSC2 by the MAP kinase regulated protein, MK2, reportedly inhibits TSC2 and leads to activation of mTOR [54]. In contrast, phosphorylation by the AMP-activated protein kinase (AMPK) on distinct residues activates TSC2 and results in repressed signaling through mTOR, suggesting that the GAP activity of TSC2 is enhanced by AMPK [55]. Until recently, the kinase that regulates AMPK was unknown. However, a recent study reports that LKB1 phosphorylates AMPK on the activating residue, Thr172, and likely represents an authentic AMPK kinase [56]. LKB1 was originally identified as a tumor suppressor that functions to limit cell growth, and is ubiquitously expressed in mammalian tissues [57,58]. Alone, LKB1 does not phosphorylate AMPK, but when complexed with two adapter proteins, STRAD and MO25, it exhibits potent AMPK activity [56]. Two isoforms (α and β) of each protein exist in human cells, and the complex of LKB1 with the α-isoform of each protein, i.e. LKB1•STRADα•MO25α, exhibits greater AMPK kinase activity compared to other permutations of the complex [56]. In addition to enhancing its AMPK kinase activity, STRAD and MO25 also target LKB1 to the cytoplasm; LKB1 normally is found primarily in the nucleus [59]. LKB has multiple phosphorylation sites and mutation of either Thr336 or Ser431 prevents LKB1 from inhibiting cell growth [60]; Thr336 is an autophosphorylation site, whereas Ser431 is phosphorylated by both PKA and p90^rsk ^[61]. These studies provide a possible mechanism by which glucagon might downregulate mTOR activity, i.e. phosphorylation of LKB1 by PKA might repress the AMPK kinase activity of LKB1. However, such an idea is still speculative at this point as the effect of LKB1 phosphorylation by PKA on its ability to phosphorylate AMPK has yet to be investigated.

## mRNA cis-acting elements mediating translational control

Changes in translation initiation can manifest as either altered translation of most or all mRNAs (i.e. global changes) or as altered translation of mRNAs encoding specific proteins. The mRNAs that encode proteins whose expression are specifically regulated through changes in mRNA translation (as opposed to changes in global mRNA translation) typically have one or more structural elements within the 5'-untranslated region (5'-UTR) that mediate translational control. Examples of such elements include multiple upstream open reading frames (uORF), internal ribosome entry sites (IRES), highly structured 5'-UTRs, terminal oligopyrimidine (TOP) tracts immediately downstream of the 5'-m^7^GTP cap, and binding domains for specific regulatory proteins (e.g. the iron-responsive element in the ferritin mRNA). Each of these elements serves to modulate the translation of a subset of mRNAs in response to various stimuli. For example, uORF elements repress the translation of most mRNAs under normal growth conditions. The translation of mRNAs bearing multiple uORFs is paradoxically enhanced in response to phosphorylation of the α-subunit of eIF2, an event that is associated with repressed translation of most mRNAs. The mechanism through which eIF2α phosphorylation enhances the translation of mRNAs containing multiple uORFs is complex and involves inhibition of the guanine nucleotide exchange activity of a second translation initiation factor, eIF2B, by phosphorylated eIF2. Examples of enhanced translation of mRNAs containing uORFs concomitant with eIF2α phosphorylation include the induction of the transcription factors ATF4 in mouse embryo fibroblasts deprived of amino acids (5) and CD36 in response to hyperglycemia (6) and the induction of the cationic amino acid transporter (CAT-1) in response to deprivation of amino acids [62] or glucose [63]. However, although enhanced translation of mRNAs with uORF sequences has been demonstrated in yeast and in cell lines, a similar phenomenon has not been demonstrated in an intact tissue.

A second 5'-UTR structure that allows preferential translation when eIF2α is phosphorylated is an IRES. An IRES allows the ribosome to bind to an internal site in the 5'-UTR and bypass the normal route of association with the mRNA, i.e. binding to the 5'-cap structure [reviewed in [64,65]]. The best characterized IRES-containing mRNA that is regulated by eIF2 phosphorylation is that encoding CAT-1 [62]. Like many IRES-containing mRNAs, the 5'-UTR of the CAT-1 mRNA has both an IRES and uORFs, and both elements are required for optimal regulation of CAT-1 mRNA translation. Thus, translation of an uORF adjacent to the IRES promotes a rearrangement of the IRES structure, resulting in its activation. However, this mechanism alone is unlikely to account completely for the enhanced translation of the CAT-1 mRNA because enhanced CAT-1 synthesis is delayed several hours after induction of eIF2α phosphorylation, suggesting that synthesis of another protein might be required for translation of the CAT-1 mRNA. Proteins that bind to IRES elements and modulate their function are referred to as IRES-transacting factors (ITAFs). Although poorly characterized, it has been suggested that ITAFs function as RNA chaperones that, upon binding to the IRES, promote refolding of the domain into the correct structure for 40S ribosome binding. Examples of ITAFs include the polypyrimidine tract binding protein (PTB) and upstream of N-ras (unr) that activate the Apaf-1 IRES [66].

Although eIF2α phosphorylation is one mechanism for enhancing the translation of mRNAs containing an IRES element(s), it is not unique. For example, during apoptosis or infection by certain types of viruses, eIF4G is cleaved. The normal function of eIF4G is to assemble the translation initiation factors eIF4A and eIF4E and the poly(A) binding protein into a complex that mediates the binding of mRNA to the 40S ribosomal subunit. Cleavage of eIF4G during apoptosis or viral infection separates the binding domain for PABP and the mRNA cap binding protein, eIF4E, from the domains that bind eIF4A and allow ribosome attachment (referred to as the middle fragment of eIF4G or M-FAG). A recent study reported that M-FAG generated in etoposide-treated cells M-FAG promotes the preferential translation of certain, but not all, IRES-containing mRNAs including Apaf-1 and death-associated protein (DAP)-5 [67]. Moreover, a number of IRES-containing mRNAs are preferentially translated under conditions that promote dephosphorylation or decreased function of eIF4E, for example when eIF4E is associated with one of the eIF4E binding proteins such as 4E-BP1. Thus, IRES function can be regulated through multiple mechanisms.

Another structural element within the 5'-UTR of some mRNAs that is involved in selective mRNA translation is an oligopyrimidine tract, referred to as a TOP sequence, immediately downstream of the 5'-cap structure [68,69]. Messages containing a TOP sequence include those encoding the ribosomal proteins, eukaryotic elongation factors-1A and 2, PABP, and eIF4G; in other words, proteins involved in protein synthesis. Thus, enhanced translation of TOP mRNAs is one mechanism for increasing ribosome biogenesis and the long-term capacity to synthesize protein. In liver of fasted rats, inhibition of mTOR by rapamycin prevents completely the leucine-induced phosphorylation of S6K1 and rpS6 as well as the increased association of TOP mRNAs with polysomes, suggesting an important role for S6K1 activation in the regulation of TOP mRNA translation [70]. Similarly, rapamycin prevents the feeding-induced increase in S6K1 phosphorylation in liver and skeletal muscle of neonatal pigs [71]. However, recent studies [72-74] suggest that activation of S6K1 may not be the only mechanism for enhancing translation of TOP mRNAs, although possible alternatives have not been identified.

Most mRNAs that are efficiently translated, e.g. GAPDH and β-actin, have 5'-UTRs that are short (<200 nt), have a low content of G and C residues, and are relatively unstructured [75]. In contrast, other mRNAs contain long, highly-structured 5'-UTRs. It isn't surprising that in order for the 40S ribosome to reach the AUG start codon of mRNAs with highly-structured 5'UTRs, the RNA helicase activity of eIF4A is essential. However, the results of a recent study suggest that both eIF4A and the eIF4A enhancer eIF4B are required for optimal translation of most mammalian mRNAs, including those such as the β-actin mRNA [76]. Although little is known about the mechanism(s) through which eIF4A and eIF4B might be regulated, eIF4B is phosphorylated on Ser422 in vitro by S6K1 and leucine-deprivation of cells in culture promotes dephosphorylation of eIF4B [77]. Thus, eIF4B phosphorylation by S6K1 provides a possible link between hormone and nutrient signaling through mTOR and eIF4A/eIF4B function.

Secondary structure may also function as a cis-acting regulatory element through the binding of specific trans-acting factors. A well-characterized example of such regulation is the modulation of ferritin and δ-aminolevulinate (ALA) synthase mRNA translation in response to changes in iron availability [78]. Both the ferritin and ALA synthase mRNAs contain hairpin structures near the 5'-end of their mRNAs, termed an iron-responsive element (IRE), that specifically binds to the IRE-binding proteins IRP1 and IRP2. Low intracellular iron enhances the IRE binding activity of IRP1 and the stability of IRP2 allowing them to bind to the IRE structure and stabilize it. Because of its proximity to the 5'-cap structure, the IREIRP1/2 complex blocks the binding of the 40S ribosome to the mRNA, thereby preventing translation of the ferritin and ALA synthase mRNAs.

## Conclusions

Nutrients, and in particular certain amino acids, play important roles in the control of gene expression through their ability to modulate the initiation phase of mRNA translation. All essential amino acids have the potential to globally regulate mRNA translation through the eIF2α kinase mGCN2. In addition, changes in eIF2α phosphorylation can selectively modulate the translation of mRNAs encoding particular proteins if the 5'UTR of the mRNA contains uORFs and/or IRES elements. Selective control of mRNA translation can also occur through changes in signaling through mTOR. Activation of S6K1 by mTOR leads to phosphorylation of rpS6 and eIF4B which is thought to promote preferential translation of TOP mRNAs and mRNAs with highly structured 5'-UTRs, respectively. In addition, mTOR phosphorylates the eIF4E binding proteins leading to enhanced assembly of the eIF4F complex. In combination with eIF4B phosphorylation, enhanced eIF4F assembly leads to preferential translation of mRNAs with highly structured 5'-UTRs. Although other amino acids have been shown to increase signaling through mTOR, leucine is arguably the most potent of the amino acids in activating the pathway.

## Authors' contributions

Both authors contributed equally to the writing of this manuscript.

## Competing interests

None declared.
